# Science surrounding the safe use of bioactive ingredients in infant formula: federal comment

**DOI:** 10.1038/s41390-023-02512-6

**Published:** 2023-02-09

**Authors:** Ashley J. Vargas, Carrie Assar, Andrew A. Bremer, Susan J. Carlson, Jeremiah Fasano, Jaime Gahche, Kimberlea Gibbs, Patricia A. Hansen, Andrea Lotze, Robin A. McKinnon, Rachel Morissette, Nancy Potischman, Kotaro Kaneko

**Affiliations:** 1 Eunice Kennedy Shrive National Institute of Child Health and Human Development, Bethesda, MD USA; 2 Center for Food Safety and Applied Nutrition, College Park, MD USA; 3 Office of Dietary Supplements, National Institutes of Health, Bethesda, MD USA

## Abstract

Less than a quarter of U.S. infants meet the federal recommendation for exclusively breastfeeding to 6 months of age, necessitating access to safe and effective infant formula. The U.S. National Institutes of Health (NIH) and Food and Drug Administration (FDA) held a public Workshop titled “Exploring the Science Surrounding the Safe Use of Bioactive Ingredients in Infant Formula: Considerations for an Assessment Framework” on September 23^rd^ and September 24^th^, 2021. Commentary from the NIH and FDA perspectives on the workshop topics is provided in this publication.

## Introduction

Human milk research has revealed a complex and dynamic biological system within human milk^1–3^. This has illuminated the possibility that biologically active (bioactive) constituents that mimic those found in human milk could be added to infant formula as new ingredients to improve child health. Although human milk is recommended as the sole source of nutrition for infants up to about 6 months of age, only about 25% of infants in the U.S. meet this objective of exclusive breastfeeding^4^, making access to a safe and effective source of nutrition provided by infant formula critical for child health. Although research on the function of these constituents is ongoing, the U.S. Food and Drug Administration (FDA) is tasked with making timely decisions on whether these ingredients can be safely added to infant formula, and if so, at what levels and combinations, and under which conditions, given the existing knowledge base. This challenge led to the partnership between the National Institutes of Health (NIH) and the FDA to host a scientific workshop describing the state of the science on the functional aspects of biologically active human milk components and analogs that could impact safety decisions.

## Background

NIH is the largest funder of biomedical research in the world. Research on infant nutrition, including infant formula, is often supported by the National Institute of Child Health and Human Development (NICHD)^5^, but is also supported by other NIH Institutes, Centers, and Offices^6^. The FDA’s Center for Food Safety and Applied Nutrition (CFSAN) regulates the safety and labeling of approximately 80% of the U.S. food supply and has pre-market regulatory authority over all infant formula (including formulas for both healthy, term infants and for those infants who require a specialized formula) and its ingredients. As a science-based public health agency, CFSAN uses robust, peer-reviewed science as much of the basis for regulatory decision making. All infant formula marketed in the U.S. must meet federal nutrient requirements^7^, as well as satisfy other regulations, including the stipulation that all ingredients added to infant formula intended for healthy, term infants are safe and suitable for use in infant formula (i.e., an approved food additive, Generally Recognized as Safe (GRAS) for such use, or authorized by prior sanction)^8^. Infant formula manufacturers must notify the FDA prior to marketing a formula with a new ingredient. Given the growing recognition of the importance of research on exposures during the first 1,000 days emerging research on the variety of human milk components and questions on their function;^1–3,9,10^ and the increasing number of new or proposed new ingredients intended for infant formula;^11^ both Agencies closely collaborated to develop a workshop to describe and discuss the state of the science on bioactive ingredients for use in infant formula.

## Pre-session to the workshop

Prior to the workshop, the NIH and FDA held a Pre-session meeting to detail the interests of both Agencies in this space, and to provide a brief background on the regulatory framework for infant formula and for evaluating the safety of food ingredients, including ingredients for use in infant formula. The Pre-session was recorded and is publicly available for viewing on the Workshop website^12^.

## Workshop overview

The joint NIH and FDA Workshop titled “Exploring the Science Surrounding the Safe Use of Bioactive Ingredients in Infant Formula: Considerations for an Assessment Framework” was held on September 23^rd^ and September 24^th^, 2021. The workshop was open to the public, completely virtual, recorded for future public viewing, and featured presentations and discussions by leading non-federal researchers. The agenda, access to the recorded workshop, and more information can be found on the meeting website^12^.

For the purposes of this workshop, only infant formula intended for use in healthy, term infants was discussed and a bioactive ingredient was defined as follows: Bioactive ingredients are synthetic or non-human milk-derived ingredients that may mimic components typically present in human milk, and that are not traditionally considered essential nutrients but are hypothesized to have physiological activity. These ingredients are intended to be added to infant formula, either alone or in combination, with awareness of the hypothesized biological activity, and may include microbiome products such as live microbes, microbial constituents, and products of microbial metabolism.

The Directors of both the NICHD and CFSAN began the workshop by affirming their commitment to infant formula-related research and its importance for public health. Leading researchers from myriad academic institutions and scientific backgrounds then spoke and discussed many topics relating to assessing the use of bioactive ingredients in infant formula for healthy, term infants (see agenda^12^). Throughout the presentations and discussions, a reoccurring theme of “we don’t know yet” was echoed by the speakers on many topics, indicating the emerging nature of human milk and infant formula bioactive ingredient research. Other themes included:

- an appreciation for the ever-increasing complexity in human milk research that is the primary basis of most research in infant formula innovations
- the variability in human milk composition and volume among mother-infant dyads and during different phases of lactation, making it difficult to determine optimum bioactive ingredient concentrations for addition to infant formula and to predict quantitative response(s) to any given bioactive milk constituent used as an infant formula ingredient
- a need for in-depth, long-term studies to determine the function and safety of bioactives for use in infant formula, but a lack of consensus on the required length of clinical study follow-up periods
- that while much is unknown about the effects of single bioactive milk constituents, even less is known about the potential for interactions between these bioactive constituents under different conditions and within an infant formula matrix, suggesting that model systems for evaluating these effects need to be expanded
- a need for the development of a more standardized approach for evaluating the function and safety of bioactive infant formula ingredients, including model systems, databases of information on infants and children, and big data analytics

Details on the viewpoints, opinions, and facts shared by speakers are available in the workshop proceedings are published here^13^.

The workshop demonstrated that much of the determination of function and safety of bioactive human milk constituents (and their analogs) as new infant formula ingredients relies upon human milk research. Separate from the human milk research needs, the workshop revealed large research gaps that are specific to assessing potential bioactive ingredients for use in infant formula. These gaps reflect technical challenges from source materials (e.g., structural and physiological evaluation of ingredients from bovine and other sources as surrogates for human milk constituents), as well as differences between human milk and infant formula matrices, both resulting in potentially different bioactivity of the ingredient/constituent. Further, the discussions identified that there is limited overlap between primary academic interests in human milk, the interests of industry and other stakeholders invested in improving alternative modes of feeding, and the interests of food safety assessors who ensure reasonable certainty of no harm for all infants regardless of potential benefit. These discussions revealed a need for close engagement of federal entities. A number of speakers also suggested using a risk-benefit type of assessment to aid in FDA’s evaluation. However, given that the regulatory framework categorizes infant formula and its ingredients as foods, not as drugs or biologics, any potential or actual benefits are not considered in FDA’s safety evaluation (see FDA resource^14^ and Pre-session recordings^12^ for more detail). Furthermore, under FDA’s safety evaluation, all ingredients must meet the reasonable certainty of no harm safety standard under the intended conditions of use. These concerns regarding evaluation within a food safety regulatory framework are still unresolved. FDA remains committed to engaging with the scientific community, through workshops like this one, to explore all avenues to ensure that all ingredients for use in infant formula are safe.

## Conclusion

It is clear from infant formula’s history^15^ that multiple entities, including researchers, pediatricians, regulators, and the infant formula industry have all contributed to making available safe and appropriate formulations for alternative infant feeding. While the decision and justification for proposing to add bioactive ingredients to infant formula are the responsibility of infant formula companies, in addition to regulation, there is a role for the federal government in supporting some research on infant formula to protect and promote public health. A better understanding on the part of researchers of FDA’s perspectives, as reflected in its regulations and guidance documents, could lead to fruitful efforts towards filling some of the research gaps identified. In parallel, NIH can provide a conduit, such as this workshop and grant opportunities, through which these interactions can take place. There are examples of successful research partnerships between NIH and FDA, for example the Tobacco Regulatory Science Program^16^. This and other models or methods could be considered by federal agencies to move infant formula research forward to support the health of our youngest Americans, if additional resources were available to support such efforts.

## References

[1] Bode L, Raman AS, Murch SH, Rollins NC, Gordon JI (2020). Understanding the mother-breastmilk-infant “triad”. Science.

[2] Christian P (2021). The need to study human milk as a biological system. Am. J. Clin. Nutr..

[3] Goldman AS (2019). Future research in the immune system of human milk. J. Pediatr..

[4] Control CfDPa 2022 Breastfeeding Among U.S. Children Born 2012-2019, CDC National Immunization Survey.

[5] NICHD 2020 NICHD Strategic Plan.

[6] Program NIoHCF 2021 The Common Fund’s Glycoscience program.

[7] Goverment USF 2022 Code of Federal Regulations—PART 107 - INFANT FORMULA. U.S. Federal Goverment.

[8] Goverment USF 2022 Code of Federal Regulations—PART 106. U.S. Federal Government.

[9] Haschke F, Haiden N, Thakkar SK (2016). Nutritive and bioactive proteins in breastmilk. Ann. Nutr. Metab..

[10] Moossavi S (2019). Composition and variation of the human milk microbiota are influenced by maternal and early-life factors. Cell Host Microbe.

[11] Administration FaD 2022 GRAS Notices.

[12] NICHD 2021 Exploring the science surrounding the safe use of biologically active ingredients in infant formula: considerations for an assessment framework. https://web.cvent.com/event/ec019daf-1ecf-4ff5-b84c-92e7bf5c3949/summary.

[13] Donovan, S. M. et al. Summary of the joint National Institutes of Health and the Food and Drug Administration workshop titled "exploring the science surrounding the safe use of bioactive ingredients in infant formula: Considerations for an assessment framework". *J Pediatr.* 2022:S0022-3476(22)01085-X. 10.1016/j.jpeds.2022.11.027. 10.1016/j.jpeds.2022.11.027 PMC10121942 36463938

[14] Administration FaD 2006 Guidance for Industry: Frequently Asked Questions about FDA’s Regulation of Infant Formula.

[15] Fomon S (2001). Infant feeding in the 20th century: formula and beikost. J. Nutr..

[16] Backinger CL, Meissner HI, Ashley DL (2016). The FDA “Deeming Rule” and tobacco regulatory research. Tob. Regul. Sci..

